# The association of plasma NT-proBNP level and progression of diabetic kidney disease

**DOI:** 10.1080/0886022X.2022.2158102

**Published:** 2023-02-23

**Authors:** Yuancheng Zhao, Lijun Zhao, Yiting Wang, Junlin Zhang, Honghong Ren, Rui Zhang, Yucheng Wu, Yutong Zou, Nanwei Tong, Fang Liu

**Affiliations:** a Division of Nephrology, West China Hospital of Sichuan University, Chengdu, Sichuan China; b Laboratory of Diabetic Kidney Disease, Centre of Diabetes and Metabolism Research, West China Hospital of Sichuan University, Chengdu, Sichuan, China; c Division of Endocrinology, West China Hospital of Sichuan University, Chengdu, Sichuan, China

**Keywords:** NT-proBNP, diabetic kidney disease, type 2 diabetes mellitus, end-stage kidney disease

## Abstract

**Aims:**

Diabetic kidney disease (DKD) is the most common cause of end-stage kidney disease (ESKD). The identification of risk factors involved in the progression of DKD to ESKD is expected to result in early detection and appropriate intervention and improve prognosis. This study aimed to explore whether plasma N-terminal pro-B-type natriuretic peptide (NT-proBNP) was associated with kidney outcomes in patients with type 2 diabetes mellitus (T2DM) and biopsy-proven DKD.

**Methods:**

Patients with biopsy-proven DKD who were followed up at West China Hospital over 12 months were enrolled. The kidney outcome was defined as progression to ESKD. The cutoff value of plasma NT-proBNP concentration was calculated by using receiver operating characteristic (ROC) curve analysis. The influence of NT-proBNP levels on kidney outcome in patients with DKD was assessed using Cox regression analysis.

**Results:**

A total of 30 (24.5%) patients reached ESKD during a median follow-up of 24.1 months. The baseline serum NT-proBNP level had a significant correlation with baseline proteinuria, kidney function, glomerular lesions, interstitial fibrosis tubular atrophy (IFTA), and arteriolar hyalinosis. Multivariate Cox regression analysis indicated that increased NT-proBNP level was significantly associated with a higher risk of progression to ESKD (HR 6.43; 95% CI (1.65–25.10, *p* = 0.007), and each 1 SD increase in LG (NT-proBNP) was also associated with a higher risk (HR 2.43; 95% CI 1.94–5.29, *p* = 0.047) of an adverse kidney outcome after adjusting for confounding factors.

**Conclusions:**

A higher level of plasma NT-proBNP predicts kidney prognosis in patients with biopsy-proven DKD. This warrants further investigation into the potential mechanisms.

## 1. Introduction

Diabetic kidney disease (DKD) is one of the most serious microvascular complications in patients with diabetes. Centers for Disease Control and Prevention (CDC) showed that approximately 44% of the patients initiating end-stage kidney disease (ESKD) treatment had diabetes ranked as the leading cause of ESKD in the United States in 2017 [1]. In addition, Afkarian et al. [2]. observed that DKD carries a 10-year mortality rate of up to 31% in patients with type 2 diabetes mellitus (T2DM). Early detection and better management of DKD in patients with T2DM may delay the progression to ESKD and improve its complications and outcomes. Although renoprotective interventions have been universally implemented to improve glycemia, blood pressure, and serum lipid regulation over the last decades, the risk of ESKD and the health burden in DKD patients are still increasing [3]. Searching for further insight into the pathogenesis and risk factors for DKD development is extremely urgent and essential for the clinical management of DKD.

ProBNP (pro-B-type natriuretic peptide) is secreted by cardiomyocytes in response to stretching and is quickly cleaved into two circulating fragments—the biologically active 32-amino acid C-terminal BNP (B-type natriuretic peptide) and the inert 76-amino acid NT-proBNP—with a 1:1 molar ratio [4]. NT-proBNP is secreted from the ventricular myocardium in response to increased myocyte stress and volume overload. Volume overload is frequently observed in patients with T2DM at high cardio-kidney risk [5]. Recently, the ADVANCE Trial indicated that NT-proBNP may help to identify patients with T2DM who are at greatest risk of microvascular complications, particularly nephropathy [6], and the CRIC Study also showed that NT-proBNP is strongly associated with CKD progression among those with and without diabetes [7]. Diabetes with rapidly worsening kidney disease is often ‘clinically’ labeled as having diabetic kidney disease (DKD), whereas in many cases, they are developing nondiabetic kidney disease (NDKD) or mixed forms (DKD + NDKD). Nondiabetic kidney disease (NDKD) and mixed forms are common in patients with CKD and diabetes (27–82.9% and 4–45.5%, respectively) [8].

Thus, this study was performed in patients with T2DM and biopsy-proven DKD 1) to explore the relationship between NT-proBNP concentration and pathological changes and 2) to explore whether NT-proBNP as a biomarker could predict kidney prognosis in patients with T2DM and biopsy-proven DKD.

## 2. Methods

### 2.1. Patient selection and study design

**Figure 1.**
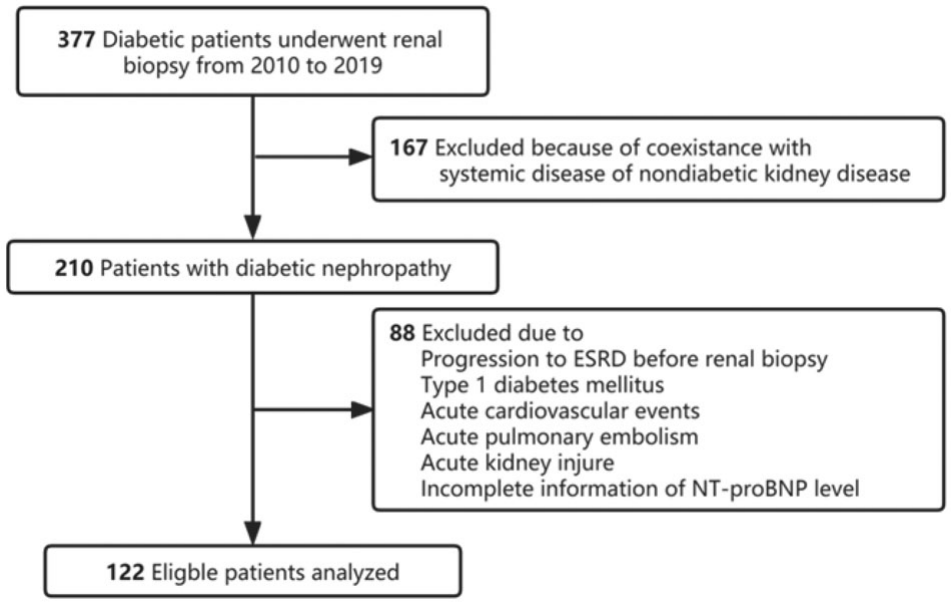
Flowchart of study participants.

To explore the relationship between plasma NT-proBNP concentration and the progression of DKD, this study included patients with T2DM and DKD who underwent kidney biopsy from 2010 to 2019 at the West China Hospital of Sichuan University. T2DM was diagnosed according to American Diabetes Association (ADA) criteria [9]. DKD was defined according to the standard published by An et al. [10] in 2015 and was diagnosed based on the Kidney Pathology Society (RPS) classification [11]. The indications for a kidney biopsy at our institution were T2DM and kidney damage, especially in T2DM patients without diabetic retinopathy, with sudden onset overt proteinuria, with obvious glomerular hematuria, or with rapidly declining kidney function [12]. Patients with coexisting nondiabetic kidney diseases (such as membranous and IgA nephropathy, systemic lupus erythematosus, ANCA-associated vasculitis), T1DM, acute cardiovascular events (such as hospitalization for heart failure, myocardial infarction, and stroke within three months before kidney biopsy) [13], acute pulmonary embolism [14], acute kidney injury [15], urosepsis [16], progression to ESKD before kidney biopsy, and patients without baseline serum NT-proBNP level information were excluded (Figure 1). All patients provided written informed consent, and this study was approved by the institutional review board at the West China Hospital of Sichuan University.

### 2.2. Measurement of plasma NT-proBNP levels

Fasting blood samples were collected from a peripheral vein into tubes containing aprotinin and ethylenediaminetetraacetic acid at the time of kidney biopsy. Plasma samples were stored at -150 °C and thawed just before testing. The plasma NT-proBNP concentration was measured with an electrochemiluminescence immunoassay kit (Roche Diagnostics, Grenzach Wyhlen, Germany) at West China Hospital of Sichuan University [17].

### 2.3. Clinical and laboratory data collection

Clinical and pathologic data, including age, sex, body mass index (BMI), systolic/diastolic blood pressure, and duration of diabetes, were obtained from electronic medical records at the time of kidney biopsy. Laboratory data at the time of biopsy were also obtained from the medical records. The Chronic Kidney Disease Epidemiology Collaboration formula was used to evaluate the estimated glomerular filtration rate (eGFR) [18]. Patient follow-up examinations were performed 2–4 times per year based on the patient’s condition.

### 2.4. Pathological characteristics

Kidney biopsy samples were prepared for light microscopy, immunofluorescence, and electron microscopy using standard procedures at West China Hospital of Sichuan University. For light microscopy examination, hematoxylin and eosin, periodic acid–Schiff, Masson’s trichrome, and periodic acid–Schiff silver methenamine were used to stain the kidney specimens. The original findings of immunofluorescence microscopy and electron microscopy were used to confirm the diagnosis of DKD. RPS glomerular classifications, interstitial fibrosis tubular atrophy (IFTA), interstitial inflammation, arteriosclerosis, and arteriolar hyalinosis were assessed and scored according to the RPS classification [11]. The pathologists were blinded to the clinical data and kidney outcome. Finally, the overall pathological risk score, the diabetic pathological score (D-score) [19], which is considered a useful DKD pathological scoring system in terms of predicting kidney outcome, was introduced. The D-score was calculated by summing the scores of all kidney pathological characteristics.

### 2.5. Kidney outcomes

The kidney outcome was defined by the progression to ESKD, which was defined as the need for chronic kidney replacement therapy or eGFR <15 mL/min/1.73 m^2^ over three months [20]. All patients enrolled in this study were followed until January 2020.

### 2.6. Statistical analysis

Continuous variables that were normally distributed were expressed as the mean and standard deviation (SD). When continuous variables were not normally distributed, they were expressed as the median and interquartile range (IQR). Categorical variables were expressed as percentages and counts. Differences in continuous variables between patients with different groups of NT-proBNP were analyzed by ANOVA or the Kruskal–Wallis H test, as appropriate. Categorical variables were compared with the chi-squared test. Spearman correlation analysis was used to analyze the association between plasma NT-proBNP levels and clinical-pathological covariates.

Based on the receiver operating characteristic (ROC) curve analysis, the values of plasma NT-proBNP level that best characterized the patients were selected. Survival curves of plasma NT-proBNP levels were obtained by Kaplan–Meier methods with a log-rank test. Univariate and multivariable Cox proportional hazard models were used to estimate the hazard ratios (HRs) for ESKD. We applied multivariable Cox proportional hazard models, including clinicopathological parameters (age, sex, SBP, history of CVD, eGFR, proteinuria, RPS classification, and IFTA). Age and sex were chosen based on biological plausibility. The clinical and pathological covariates were selected as potential confounders because of their significance in univariate analysis or association with ESKD in previous studies [10,21]. Parameters with *p* < 0.05 in the adjusted model were considered to be significant predictors of prognosis. All statistical analyses were performed using IBM SPSS statistics (version 23, Chicago, IL, USA). Statistical significance was accepted at *p* < 0.05.

## 3. Results

### 3.1. Baseline clinical characteristics according to the plasma NT-proBNP concentrations

The baseline clinical and laboratory characteristics of the patients are displayed in Table 1. The mean age of the patients was 51.2 years, and 82 (67.2%) patients were men. The mean BMI of the patients was 25.5 kg/m^2^, the mean duration of diabetes was 112.4 months, the mean SBP was 141.6 mmHg, the mean serum albumin was 36.9 g/L, and the mean hemoglobin was 125.3 g/L. The median baseline eGFR was 59.0 mL/min/1.73 m^2^, the median 24-h proteinuria was 3.0 g/d, and the median HbA1c was 7.3%. Thirteen (10.6%) patients had a history of CVD at baseline.

**Table 1.** The clinical characteristics according to the plasma NT-proBNP concentrations.

Parameters	All (*n* = 122)	Group 1 (*n* = 54)	Group 2 (*n* = 34)	Group 3 (*n* = 34)	*p*
Age (years)	51.2 ± 10.6	47.9 ± 10.8	51.2 ± 8.9	56.6 ± 9.9	0.001
Body mass index (kg/m^2^)	25.5 ± 3.0	25.5 ± 2.6	25.7 ± 3.3	25.3 ± 3.1	0.873
Gender (Male, %)	82 (67.2)	39 (72.2)	21 (61.8)	22 (64.7)	0.557
Duration of diabetes (months)	102 (48–168)	96 (45–159)	102 (57.5–147.0)	120 (36–180)	0.909
Hypertension (%)	74 (60.6)	28 (51.9)	20 (58.8)	26 (76.5)	0.064
SBP (mm Hg)	141.6 ± 25.1	136.2 ± 24.8	140.8 ± 23.2	150.9 ± 25.5	0.026
DBP (mm Hg)	84 (74-92)	84 (74.0-90.0)	84 (71.5-93.5)	84.5 (76.2-95.0)	0.970
24-h proteinuria (g/d)	3.3 (1.2–6.4)	1.75 (0.5–3.6)	3.3 (2.1–5.9)	6.9 (5.1–9.7)	<0.001
Serum creatinine (umol/L)	112.0 (75.0–144.0)	86.5 (65.5–125.5)	102.7 (74.2–141.7)	141.0 (114.0–164.0)	<0.001
e-GFR (mL/min/1.73 m^2^)	59.0 (45.5–94.2)	83.3 (54.1–109.2)	67.0 (51.4–94.2)	43.0 (36.3–52.2)	<0.001
BUN (mg/dl)	7.4 (5.6–10.0)	6.5 (5.2–9.4)	7.3 (5.2–9.5)	9.0 (7.4–11.9)	0.002
Serum albumin (g/L)	36.9 ± 7.1	40.5 ± 5.6	36.5 ± 6.3	31.4 ± 6.3	<0.001
FBS (mmol/L)	7.9 (6.2–10.4)	8.3 (6.3–10.8)	7.5 (5.5–9.5)	7.9 (6.5–9.4)	0.359
HbA1c (%)	7.3 (6.5–8.6)	7.5 (6.7–8.2)	6.9 (6.6–9.0)	7.1 (6.4–9.4)	0.588
Triglyceride (mmol/L)	1.9 (1.4–2.5)	1.95 (1.37–2.80)	1.90 (1.40–2.50)	1.75 (1.27–2.22)	0.553
Total cholesterol (mmol/L)	4.8 (4.1–5.9)	4.60 (3.60–5.45)	5.15 (4.17–5.95)	5.2 (4.2–6.7)	0.125
Uric acid (mmol/L)	390.4 ± 80.8	397.4 ± 92.6	400.9 ± 68.5	368.7 ± 69.5	0.182
HDL cholesterol (mmol/L)	1.1 (0.9–1.4)	1.10 (0.90–1.30)	1.10 (0.90–1.50)	1.20 (1.10–1.40)	0.744
LDL cholesterol (mmol/L)	2.9 ± 1.2	2.63 ± 1.05	2.83 ± 1.10	3.32 ± 1.32	0.025
Hemoglobin (g/L)	125.3 ± 27.1	135.8 ± 27.8	122.9 ± 25.5	110.8 ± 19.9	<0.001
History of CVD	13 (10.7)	1 (1.9)	2 (5.9)	10 (29.4)	<0.001
Anti-hypertension drugs					
α-blockers	24 (19.7)	4 (7.4)	8 (23.5)	12 (35.3)	0.005
β-blockers	34 (27.9)	6 (11.1)	8 (23.5)	20 (58.8)	<0.001
CCB	61 (50)	15 (27.8)	22 (64.7)	24 (70.6）	<0.001
ACEI	18 (14.8)	5 (9.3)	8 (23.5)	5 (14.7)	0.185
ARB	95 (77.9)	43 (79.6)	29 (85.3)	23 (67.6)	0.197
Diuretics	22 (18.0)	4 (7.4)	5 (14.7)	13 (38.2)	0.001
Lipid-lowering therapy					
Statin	70 (57.9)	27 (50)	22 (64.7)	21 (63.6)	0.29
Baseline glucose-lowering therapies					
Metformin	47 (38.8)	30 (55.6)	11 (32.4)	6 (18.2)	0.002
Sulfonylurea	7 (5.7)	6 (11.1)	0 (0)	1 (2.9)	0.073
Dipeptidyl peptidase-4 inhibitor	42 (34.4)	20 (37)	12 (35.3)	10 (29.4)	0.758
Insulin	84 (68.9)	32 (59.3)	25 (73.5)	27 (79.4)	0.109

SBP: systolic blood pressure; DBP: diastolic blood pressure; e-GFR: estimated glomerular filtration rate; FBS: fasting blood sugar; CVD: cardiovascular disease; CCB: calcium channel blockers; ACEI: angiotensin-converting enzyme inhibitor; ARB: angiotensin II receptor blocker. Data are presented as the mean ± standard, the median or counts and percentages; Differences between groups were analyzed using the ANOVA, the Kruskal–Wallis *H* test or the chi-square test, as appropriate.

**Figure 2.**
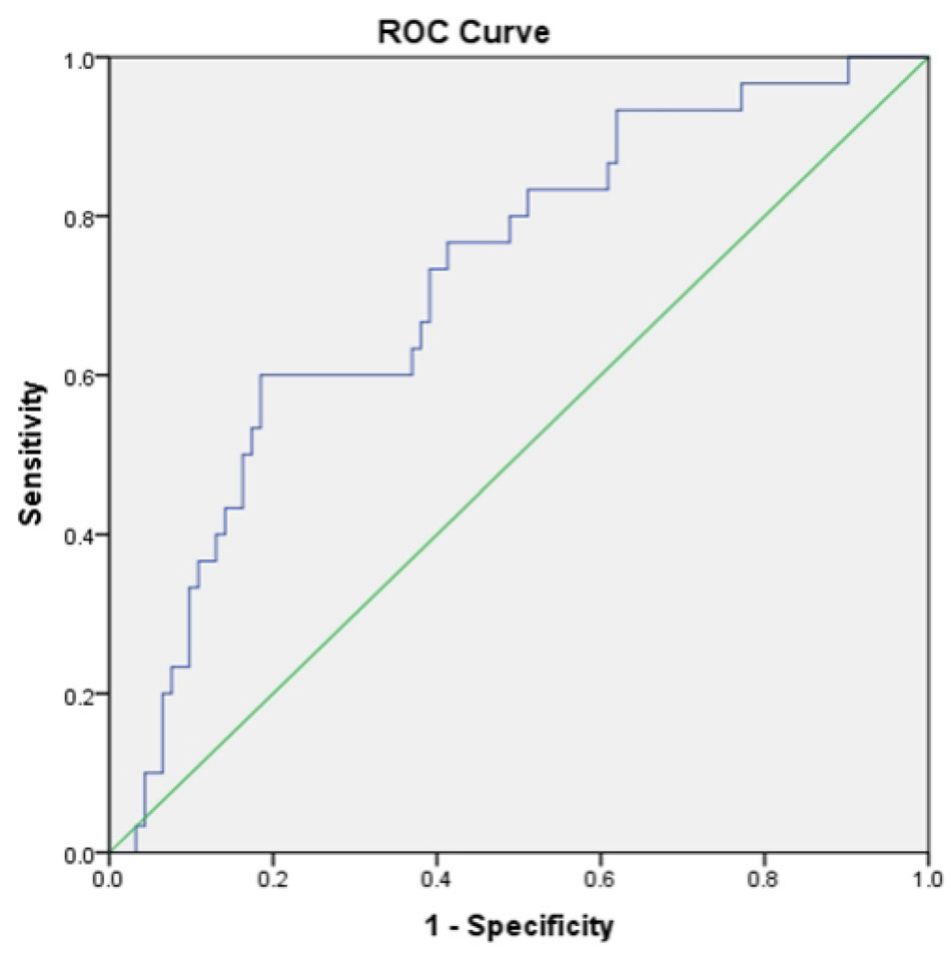
ROC curve analysis of the NT-proBNP level for prediction of progression of diabetic kidney disease.

The area under the ROC curve for NT-proBNP at baseline for prediction of progression of diabetic kidney disease was 0.72 [95% CI (0.618,0.822), *p* < 0.001] **(**Figure 2). A cutoff value in the ROC curve analysis for baseline NT-proBNP was 416.2 pg/ml, which had a sensitivity of 60.0% and a specificity of 81.5% for the prediction of kidney outcome.

All patients in this study were divided into three groups according to the normal level and cutoff value of baseline plasma NT-proBNP concentration: Group 1 (normal level): ≤125 pg/mL (*n* = 54); Group 2: 125–416 pg/mL (*n* = 34); and Group 3: >416 pg/mL (*n* = 34). Compared with patients in group 1, patients in group 2 and group 3 had a lower eGFR slope (Supplement Table 1) and eGFR, hemoglobin, and serum albumin levels; however, they were older and had higher SBP, serum creatinine, BUN levels, and proteinuria. There were no differences in sex distribution, BMI, duration of diabetes, DBP, baseline fasting plasma glucose, HbA1c, serum total cholesterol, uric acid, high-density lipoprotein cholesterol, or low-density lipoprotein cholesterol concentrations among the three groups. The baseline clinical characteristics of the three groups divided by the tertile of plasma NT-proBNP concentrations are shown in Supplement Table 2.

### 3.2. Baseline pathological characteristics according to the plasma NT-proBNP concentrations

According to the RPS classification, severe glomerular lesions (class III + IV) of group 1, group 2, and group 3 were observed in 18 (33.3%), 23 (67.7%), and 22 (64.7%) patients, respectively. The severe interstitial fibrosis and tubular atrophy (IFTA) scores (scores 2 and 3) of groups 1, 2, and 3 were observed in 19 (35.2%), 16 (47.1%), and 23 (67.7%) patients, respectively. The severe interstitial inflammation scores (score 2) of groups 1, 2, and 3 were observed in 8 (14.8%), 7 (20.6%), and 11 (32.4%) patients, respectively. Among 122 patients, arteriolar hyalinosis was absent in 12 patients (9.8%). At least one area of arteriolar hyalinosis (scored 1) was found in 77 (63.2%) patients. More than one area of arteriolar hyalinosis (scored as 2) was found in 33 patients (27.0%). Regarding arteriosclerosis changes, 16 (13.1%) had no intimal thickening (scored as 0), 59 (48.4%) had intimal thickening less than the thickness of the media (scored as 1), and 47 (38.5%) had severe arteriosclerosis (scored as 2). Compared with patients in group 1, the patients in groups 2 and 3 had severe RPS glomerular classification, IFTA, interstitial inflammation, arteriosclerosis, or arteriolar hyalinosis and had high kidney pathological scores (Table 2).

**Table 2.** Baseline pathological characteristics according to the plasma NT-proBNP concentrations.

Pathological lesions	All (*n* = 122)	Group 1 (*n* = 54)	Group 2 (*n* = 34)	Group 3 (*n* = 34)		*p* value*	Correlation coefficient (*r*)	*p* value#
RPS classification						0.002	0.30	0.001
I	10 (8.2)	10 (18.5)	0 (0)	0 (0)	0			
Iia	32 (26.2)	22 (40.7)	7 (20.6)	3 (8.8)	3			
Iib	17 (13.9)	12 (22.2)	4 (11.8)	1 (2.9)	4			
III	47 (38.5)	10 (18.5)	19 (55.9)	18 (52.9)	6			
IV	16 (13.1)	0 (11.1)	4 (11.8)	12 (35.3)	6			
IFTA						0.002	0.34	<0.001
0	6 (4.9)	6 (11.1)	0 (0)	0 (0)	0			
1	58 (47.5)	29 (53.7)	18 (52.9)	11 (32.4)	7			
2	48 (39.3)	18 (33.3)	11 (32.4)	19 (55.9)	9			
3	10 (8.2)	1 (1.9)	5 (14.7)	4 (11.8)	11			
Interstitial inflammation						0.248	0.23	0.012
0	3 (2.5)	2 (3.7)	1 (2.9)	0 (0)	0			
1	93 (76.2)	44 (81.5)	26 (76.5)	23 (67.6)	3			
2	26 (21.3)	8 (14.8)	7 (20.6)	11 (32.4)	4			
Arteriolar hyalinosis						0.001	0.35	<0.001
0	12 (9.8)	11 (20.4)	1 (2.9)	0 (0)	0			
1	77 (63.2)	33 (61.1)	24 (70.6)	20 (58.8)	0			
2	33 (27.0)	10 (18.5)	9 (26.5)	14 (41.2)	3			
Arteriosclerosis						0.023	0.30	0.001
0	16 (13.1)	13 (24.1)	2 (5.9)	1 (2.9)	0			
1	59 (48.4)	24 (44.4)	18 (52.9)	17 (50.0)	0			
2	47 (38.5)	17 (31.5)	14 (41.2)	16 (47.1)	1			
D-Score						<0.001	0.42	<0.001
≤14	31 (25.4)	31 (57.4)	0 (0)	0 (0)				
15–18	48 (39.3)	23 (42.6)	25 (73.5)	0 (0)				
19–21	30 (24.6)	0 (0)	9 (26.5)	21 (61.8)				
22–25	13 (10.7)	0 (0)	0 (0)	13 (38.2)				

IFTA: interstitial fibrosis and tubular atrophy; RPS classification: Renal Pathology Society classification; D-score: diabetic renal pathological score.

Correlations between the plasma NT-proBNP level and histopathological findings.

*Kruskal–Wallis H test, ^#^Spearman’s correlation analysis. A two-tailed *p* < 0.05 was considered statistically significance.

### 3.3. Associations between NT-proBNP concentration and clinical or pathological covariates

Spearman correlation analyses were performed to analyze the association between NT-proBNP and clinical characteristics (Table 3). The results showed that baseline NT-proBNP was significantly associated with 24-h proteinuria and kidney function and moderately associated with age, hypertension, systolic blood pressure, and BUN. Regarding the pathological findings, the baseline plasma NT-proBNP was significantly associated with RPS glomerular classifications, IFTA, and arteriolar hyalinosis and moderately associated with interstitial inflammation and arteriosclerosis.

**Table 3.** Associations between NT-proBNP concentration and clinical covariates.

Parameters	Correlation coefficient (*r)*	*p value*
Age (years)	0.310	0.001
SBP (mm Hg)	0.264	0.003
24-h proteinuria (g/d)	0.552	<0.001
e-GFR (mL/min/1.73 m^2^)	−0.516	<0.001
BUN (mmol/L)	0.381	<0.001

Spearman’s correlation analysis.

### 3.4. Risk of progression to ESKD according to baseline plasma NT-proBNP concentrations

**Figure 3.**
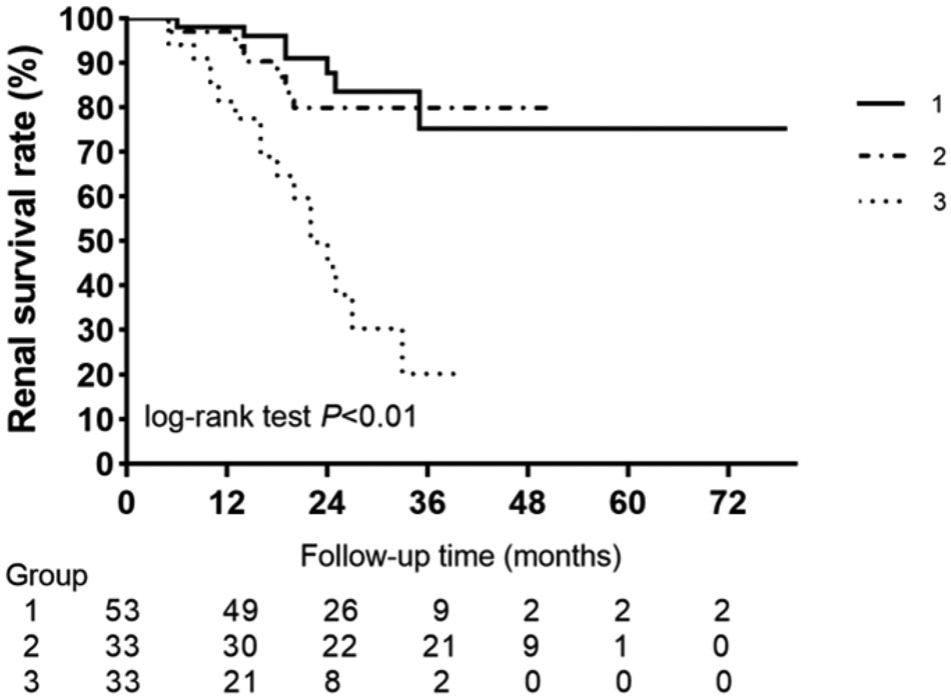
Kaplan–Meier curves of renal survival rate in patients with different plasma NT-proBNP concentrations. Log rank *p* = 0.001.

A total of 30 (24.5%) patients reached ESKD during a median follow-up of 24.1 months. The percentages of patients in group 1 (normal level), group 2 (high), and group 3 (higher) who progressed to ESKD were 7 (13%), 6 (17.6%), and 17 (50%), respectively. Figure 3 shows the survival curves of groups 1, 2, and 3 by Kaplan–Meier methods. The results showed that the baseline plasma NT-proBNP concentrations were significant for ESKD (log-rank test *p* < 0.001). The results of univariable and multivariable Cox proportional hazard analyses are presented in Table 4. Adjusted for sex, age, SBP, history of CVD, baseline eGFR, proteinuria, RPS classification, and IFTA **(Model 3)**, the upper tertile NT-proBNP level group (Group 3) experienced a higher risk of ESKD (HR 6.43; 95% CI 1.65–25.10, *p* = 0.007). Each 1 SD increase in LG (NT-proBNP) was also associated with a higher risk (HR 2.43; 95% CI 1.94–5.29, *p* = 0.047) of adverse kidney outcomes after adjusting for the abovementioned confounding factors (**Model 3**). Supplement Table 3 shows the association among the kidney outcomes and plasma NT-proBNP concentrations that were divided into three groups by the tertile of NT-proBNP concentrations.

**Table 4.** Univariable and multivariable Cox proportional hazard analysis. Associations between NT-proBNP level and renal outcomes.

	Plasma NT-proBNP, median (range) (pg/ml)	Hazard ratios (95% confidence interval) & *p* value			
		Unadjusted	Model 1	Model 2	Model 3
Per 1 SD LG (NT-proBNP)	2.29 ± 0.68	2.59 (1.57–4.29) *p* < 0.001	3.46 (1.97–6.06) *p* < 0.001	2.04 (1.09–3.83) *p* = 0.026	2.43 (1.9–45.29) *p* = 0.047
Group 1	58.1 (28.1–95.5)	Reference	Reference	Reference	Reference
Group 2	232.2 (170.7–306.8)	1.18 (0.39–3.52) *p* = 0.776	1.62 (0.52–5.04) *p* = 0.408	1.62 (0.48–5.42) *p* = 0.438	1.90 (0.50–7.17) *p* = 0.344
Group 3	1011.0 (650.9–1787.7)	5.89 (2.43–14.28) *p* < 0.001	10.11 (3.65–27.9) *p* < 0.001	4.89 (1.70–14.10) *p* = 0.003	6.43 (1.65–25.10) *p* = 0.007

Univariable and multivariable cox proportional hazard analysis. Associations between NT-proBNP level and renal outcomes. Model 1 adjusted for baseline age, gender, SBP, the history of CVD. Model 2 adjusted for covariates in model 1 plus e-GFR and proteinuria. Model 3 adjusted for covariates in model 2 plus RPS classification and IFTA. CI: confidence interval; SBP: systolic blood pressure; CVD: cardiovascular disease; e-GFR: estimated glomerular filtration rate; RPS: penal pathology society glomerular classification; IFTA: interstitial fibrosis tubular atrophy.

## 4. Discussion

In this longitudinal observational analysis of 122 patients with T2DM and biopsy-proven DKD, we found that the baseline plasma NT-proBNP levels were negatively correlated with eGFR and positively correlated with 24-h proteinuria and kidney pathological damage. Compared with patients with normal serum NT-proBNP levels (Group 1), patients in the upper tertile serum NT-proBNP level group (Group 3) had a significantly lower cumulative survival rate (Figure 2) and were associated with a higher risk of subsequent ESKD (HR = 6.43, 95% [CI] 1.65–25.10, *p* = 0.007), even after adjusting for relevant confounding factors (Table 4). Our observations that plasma NT-proBNP levels are a noninvasive marker associated with adverse kidney outcomes may help nephrologists further manage DKD.

Previous studies [22,23] found a positive correlation between NT-proBNP concentrations and the severity of proteinuria in patients with T2DM. Other studies [24,25] showed that NT-proBNP concentrations were negatively correlated with baseline eGFR and positively correlated with kidney function decline in patients with CKD. This is consistent with the results of our study. A possible reason is that NT-proBNP is only excreted from the kidney [4]. When patients have high NT-proBNP concentrations, it is suggested that the kidney has been impaired, as evidenced by increased proteinuria and decreased eGFR. In addition, some studies [13,26] have shown that NT-proBNP is strongly associated with hypertension and cardiovascular events. In this study, patients in the high NT-proBNP concentration group (Group 3) had a higher baseline SBP and a history of cardiovascular events. Therefore, patients in group 3 would be more likely to use antihypertensive drugs such as alpha-blockers, beta-blockers, CCB, and diuretics.

This study found a correlation between plasma NT-proBNP and kidney pathology changes and severe kidney pathological lesions, including severe glomerular lesions, severe IFTA, severe interstitial inflammation, severe arteriolar hyalinosis, and arteriosclerosis, mainly occurring in patients with a high level of NT-proBNP. BNP and NT-proBNP, mainly secreted from cardiomyocytes at a 1:1 molar ratio, may not only participate in fluid balance by natriuresis and vasodilation but also play an important role against the overactivation of the renin-angiotensin-aldosterone system (RAAS) and sympathetic nervous system through the NPR-A/cGMP/PKG pathway [27]. Additionally, BNP was reported to promote natriuresis and diuresis by relaxing mesangial cells, modulating tubule-glomerular feedback, and water-channel protein aquaporin-2 (AQP2) translocation [27]. It has been considered that the overactivity of the RAAS and sodium and water retention have a central role in the pathogenesis and progression of diabetic kidney disease [28]. In the early stages of DKD progression, BNP can promote natriuresis and dieresis and inhibit the overactivity of the RAAS and central nervous system (SNS) to preserve kidney structure and function. With the further deterioration of kidney damage, however, more BNP would be secreted to counterinteract against the negative impact of overactivity of SNS and RAAS and sodium and water retention in the kidney. Therefore, a high level of BNP or NT-proBNP may indicate that there is severe kidney damage in patients with DKD. However, the underlying mechanism necessitates further research to clarify.

NT-proBNP has already been proven to be a prognostic marker for patients with CKD [29,30], which includes both diabetes-related CKD and nondiabetic CKD. Amanda et al. [7] reported that plasma NT-proBNP levels were a risk factor for adverse kidney outcomes (the composite of halving eGFR or initiating kidney replacement therapy) in nondiabetic CKD patients (HR ≥ 1.5) and in diabetic CKD patients (HR ≥ 2.0) among 3379 CKD participants (47% participants with concomitant diabetes). In addition, another study [30] found that higher levels of NT-proBNP were independently associated with higher rates of ESKD and death among patients with T2DM. After adjusting for baseline eGFR, proteinuria, and other known predictors of CKD progression, such as hemoglobin and albumin, the baseline NT-proBNP remained independently associated with ESKD. All of the above studies suggest that plasma NT-proBNP may be a prognostic marker for diabetes-related CKD patients. However, nondiabetic kidney disease (NDRD) is common (27–82.9%) [8] among patients with diabetes and CKD undergoing kidney biopsy. Thus, our findings in patients with the biopsy-based diagnosis of DKD may be more justified.

In a previous study [31], a machine learning algorithm was used to develop and validate a predictive model for the risk of ESKD in patients with diabetic nephropathy, with a random forest algorithm identifying five major factors: cystatin-C, serum albumin, hemoglobin, 24-h urine urinary total protein, and eGFR (AUC 0.90 and ACC 82.65%). Zhao et al. [32] previously used machine learning algorithms to develop and validate a predictive model for the risk of kidney failure in patients with DKD, with a nomogram that included five factors: hemoglobin, NLR, serum cystatin C, eGFR, and 24-h urine protein (C-statistic 0.863). However, neither of these two studies had data on baseline plasma BNP or NT-proBNP concentrations. Thus, BNP or NT-proBNP was not available in the final model. In addition, Zhao et al. [33] also used machine learning algorithms to develop and validate a predictive model for the risk of ESKD in patients with DKD in another study, with a clinical-pathological model including cystatin C, eGFR, BNP, Log ACR, pathological grade and renin-angiotensin system (C-statistics 0.865). This suggested that BNP may predict the risk of ESKD in patients with DKD and improve the C-statistics of the predictive model. This is consistent with our findings and further shows the predictive role of NT-proBNP on DKD prognosis. This result may indicate that clinicians should take more aggressive and effective measures to prevent the progression of DKD with an increased level of NT-proBNP.

Recently, cardio-kidney syndrome, often defined as a bidirectional association between kidney disease and cardiovascular disease (CVD), has been broadly recognized [34]. There are several plausible mechanisms between cardiovascular and kidney disease involving neurohormonal activation, inflammation, oxidative stress, endothelial dysfunction, and anemia [35]. Given the role of NT-proBNP in terms of the heart and kidney, it may become a common marker for kidney and cardiovascular disease. This warrants further investigation into the role of NT-proBNP in cardio-kidney syndrome.

The KDIGO 2020 clinical practice guidelines for diabetic kidney disease [36] recommend that most patients with T2DM, CKD, and eGFR ≥ 30 mL/min per 1.73 m^2^ would benefit from treatment with both metformin and a sodium-glucose cotransporter-2 inhibitor (SGLT2i) to reduce the risk of cardiovascular events and kidney outcomes. Glucagon-like peptide-1 receptor agonist (GLP-1 RA) is generally preferred to manage glycemia when SGLT2i are contraindicated, especially in patients with eGFR < 30 mL/min per 1.73 m^2^. Therefore, in patients with diabetic kidney disease and high plasma NT-proBNP concentrations, especially those with NT-proBNP > 416 pg/mL and eGFR > 30 mL/min per 1.73 m^2^, almost all patients should be actively treated with SGLT2i to improve their cardio-kidney outcomes. GLP-1 RA should be considered when SGLT2i are contraindicated, especially in patients with NT-proBNP > 416 pg/mL and eGFR < 30 mL/min per 1.73 m^2^. Unfortunately, the 122 patients in this study were not prescribed SGLT2i and GLP-1 RA, as the drug was not available in China before the completion of this study.

This study, for the first time, revealed that the plasma NT-proBNP level was significantly associated with the progression of DKD. Of course, a few limitations in this study should be noted. First, it was a retrospective cohort study; therefore, selection bias was inevitable. Kidney biopsy is an invasive procedure, resulting in a proportion of patients being reluctant to undergo kidney biopsy, which contributes to the limited sample size and mismatched baseline data between groups in this study. However, the HRs were still significant after important confounding factors were adjusted in multivariable Cox analysis, which shows that the results in this study are still reliable. Second, the sample size was limited. Third, the severity of the patient’s condition varies when performing a kidney pathology biopsy. Fourth, the measurement of plasma NT-proBNP level was only measured once at baseline, and sequential measurements during the follow-up may help to further investigate its association with diabetic ESKD. Fifth, during follow-up, NT-pro-BNP levels did not correlate with HbA1c levels, insulin usage, or the fasting glucose levels of the patients with T2DM or DKD. Finally, we did not control therapeutic interventions (especially antidiabetic drugs with nephroprotective effects) during follow-up, which may be confounders to the results. In summary, our findings provide evidence that NT-proBNP levels can predict kidney prognosis in patients with T2DM and biopsy-proven DKD.

## 5. Conclusion

In summary, our findings provide evidence that NT-ProBNP levels can predict kidney prognosis in patients with T2DM and biopsy-proven DKD.

## Supplementary Material

Supplemental Material

## Data Availability

The datasets generated during and analyzed during the current study are available from the corresponding author upon reasonable request.
