# Supplementary material for: The association of plasma NT-proBNP level and progression of diabetic kidney disease
Source: Ren Fail. 2023 Feb 23;45(1):2158102. doi: 10.1080/0886022X.2022.2158102 (PMC9970255; doi:10.1080/0886022X.2022.2158102)
Supplement: Supplemental Material [file IRNF_A_2158102_SM7776.pdf]

Supplement table 1. The eGFR slope and renal outcome according to the baseline NT-proBNP concentrations.

parameters	All (n = 122)	Group1 (n = 54)	Group2 (n = 34)	Group3 (n = 34)	p
NT-proBNP (pg/ml)	165.0 (65.7-524.5)	59.1 (28.1-95.1)	227.1 (166.8-304.7)	1267.0 (656.2-2233.2)	＜0.001
Progressed to ESRD (%)	30 (24.6)	7 (13.0)	6 (17.6)	17 (50)	＜0.001
eGFR slope (mL/min/1.73/m2/year)	-4.5 (-7.4, -1.6)	-3.8 (-7.9, -0.3)	-7.3 (-11.8, -2.8)	-11.0 (-14.7, -7.4)	＜0.001

Note:Data are presented as the median; Differences between groups were analyzed using a simple linear model.ESRD, end-stage renal disease.

All patients were divided into three groups according to the normal level and cutoff value of baseline plasma NT-ProBNP concentration: Group 1 (normal level) : ≤125 pg/mL (n = 54); Group 2: 125-416pg/mL (n = 34); and Group 3: ＞416 pg/mL (n = 34).

Information Classification: General

Supplement Table 2. Baseline clinical characteristics according to the tertile NT-proBNP concentrations.

parameters	All (n = 122)	T1 (n = 41)	T2 (n = 41)	T3 (n = 40)	p
Age (years)	51.2 ± 10.6	47.3 ± 11.6	50.7 ± 9.1	55.8 ± 9.6	0.001
Body mass index (kg/m2)	25.5 ± 3.0	25.6 ± 2.3	25.4 ± 3.4	25.5 ± 3.1	0.984
Gender (Male, %)	82 (67.2)	29 (70.7)	25 (61.0)	28 (70.0)	0.578
Duration of diabetes (months)	102 (48-168)	96 (42-150)	108 (55-162)	108 (36-180)	0.901
Hypertension (%)	67 (54.9)	18 (43.9)	21 (51.2)	28 (70.0)	0.052
SBP (mm Hg)	141.6 ± 25.1	138.2 ± 25.9	134.5 ± 23.0	148.1 ± 25.8	0.135
DBP (mm Hg)	84 (74-92)	84 (75.5-90.5)	86 (73.0-91.5)	84.0 (74.5-95.0)	0.993
24-h proteinuria (g/d)	3.3 (1.2-6.4)	1.27 (0.5-3.7)	2.8 (1.5-4.4)	6.7 (4.1-9.7)	＜0.001
e-GFR (mL/min/1.73 m2)	59.0 (45.5-94.2)	80.7 (51.1-112.3)	79.8 (52.9-98.0)	45.0 (36.6-54.8)	＜0.001
Serum creatinine (umol/L)	112.0 (75.0-144.0)	89.0 (64.0-126.0)	82.0 (72.5-126.5)	141.5 (112-162.7)	＜0.001
BUN (mg/dl)	7.4 (5.6-10.0)	6.7 (5.2-9.7)	6.7 (5.1-8.7)	8.9 (7.1-11.1)	0.002
Serum albumin (g/L)	36.9 ± 7.1	41.3 ± 5.6	37.6 ± 5.6	31.5 ± 6.2	＜0.001
FBS (mmol/L)	7.9 (6.2-10.4)	8.3 (6.3-11.2)	7.4 (5.6-9.5)	7.9 (6.9-9.7)	0.254
HbA1c (%)	7.3 (6.5-8.6)	7.5 (6.7-8.4)	7.0 (6.4-8.5)	7.3 (6.5-9.4)	0.824
Triglyceride (mmol/L)	1.9 (1.4-2.5)	2.10 (1.55-3.60)	1.80 (1.40-2.35)	1.75 (1.30-2.27)	0.093
Total cholesterol (mmol/L)	4.8 (4.1-5.9)	4.80 (3.60-5.95)	4.70 (4.10-5.55)	5.20 (4.20-6.47)	0.415
Uric acid (mmol/L)	390.4 ± 80.8	402.8 ± 93.6	394.8 ± 77.6	373.2 ± 68.1	0.237
HDL cholesterol (mmol/L)	1.1 (0.9-1.4)	1.10 (0.90-1.30)	1.20 (0.90-1.50)	1.15 (1.10-1.40)	0.095
LDL cholestero (mmol/L)	2.9 ± 1.2	2.72 ± 1.12	2.68 ± 1.07	3.24 ± 1.25	0.052
Hemoglobin (g/L)	125.3 ± 27.1	137.7 ± 29.6	122.5 ± 24.5	115.3 ± 22.2	0.001
Progressed to ESRD (%)	30 (24.6)	5 (12.2)	7 (17.1)	18 (45.0)	0.001
NT-proBNP (pg/ml)	165.0 (65.7-524.5)	40.9 (26.1-68.0)	166.2 (121.7-238.7)	911.0 (524.6-1921.0)	＜0.001
History of CVD	13 (10.7)	3 (7.3)	2 (4.9)	10 (25.0)	0.011
Anti-hypertension drugs					
α-blockers	24 (19.7)	3 (7.3)	8 (19.5)	13 (32.5)	0.017
β-blockers	34 (27.9)	3 (7.3)	10 (24.4)	21 (52.5)	＜0.001
CCB	61 (50)	12 (29.3)	22 (53.7)	27 (67.5）	0.002

ACEI	18 (14.8)	5 (12.2)	8 (19.5)	5 (12.5)	0.573
ARB	95 (77.9)	31 (75.6)	35 (85.4)	29 (72.5)	0.345
Diuretics	22 (18.0)	4 (9.8)	5 (12.2)	13 (32.5)	0.014
Glucose-lowering therapies					
Metformin	47 (38.8)	24 (58.5)	15 (36.6)	8 (20.5)	0.002
Sulfonylurea	7 (5.7)	5 (12.2)	1 (2.4)	1 (2.5)	0.108
Dipeptidyl peptidase-4 inhibitor	42 (34.4)	17 (41.5)	12 (29.3)	13 (32.5)	0.485
Insulin	84 (68.9)	23 (56.1)	29 (70.7)	32 (80.0)	0.109
Lipid-lowering treatment					
Statin	70 (57.9)	21 (51.2)	25 (61.0)	24 (61.5)	0.571

Note: SBP, systolic blood pressure; DBP, diastolic blood pressure; e-GFR, estimated glomerular filtration rate; FBS, fasting blood sugar; ESRD, end-stage renal disease; CVD, Cardiovascular disease; CCB, Calcium channel blockers; ACEI, angiotensin-converting enzyme inhibitor; ARB, angiotensin II receptor blocker. Data are presented as the mean ± standard, the median or counts and percentages; Differences between groups were analyzed using the ANOVA, the Kruskal–Wallis H test or the chi-square test, as appropriate.

According to the tertile, NT-proBNP was divided into 3 groups: T1: ≤94.7 pg/ml, n1 = 41; T2: >94.7 pg/ml, ≤325.6 pg/ml, n2 = 41; T3 >325.6 pg/ml, n3 = 40.

Supplement table 3. Univariable and multivariable Cox proportional hazard analysis. Associations among the NT-proBNP and renal outcomes.

	Plasma NT-proBNP, median (range) (pg/ml)	Hazard ratios (95% confidence interval) & p value			
		Unadjusted	Model 1	Model 2	Model 3
T1	40.9 (26.1-68.0)	Reference	Reference	Reference	Reference
T2	166.2 (121.7-238.7)	1.18 (0.39-3.52)<br>P = 0.776	1.85 (0.55-6.20)<br>P = 0.317	2.79 (0.74-10.55)<br>P = 0.131	3.18 (0.70-14.39)<br>P = 0.132
T3	911.0 (524.6-1921.0)	5.89 (2.43-14.28)<br>P＜0.001	9.07 (2.98-27.63)<br>P＜0.001	4.86 (1.48-16.01)<br>P = 0.009	5.79 (1.34-25.00)<br>P = 0.019

Note: Univariable and multivariable cox proportional hazard analysis. Associations between NT-proBNP level and renal outcomes. Model 1 adjusted for baseline age, gender, SBP and the history of CVD. Model 2 adjusted for covariates in model 1 plus e-GFR and proteinuria. Model 3 adjusted for covariates in model 2 plus RPS classfication and IFTA. CI, confidence interval. SBP, systolic blood pressure; CVD, cardiovascular disease; e-GFR, estimated glomerular filtration rate; RPS, Renal Pathology Society glomerular classification; IFTA, interstitial fibrosis tubular atrophy.

According to the tertile, NT-proBNP was divided into 3 groups: T1: ≤94.7 pg/ml, n1 = 41; T2: >94.7 pg/ml, ≤325.6 pg/ml, n2 = 41; T3 >325.6 pg/ml, n3 = 40.

Information Classification: General

Supplement table 4. Univariable and multivariable cox proportional hazard analysis. Associations among the continuous NT-proBNP and renal outcomes.

	Plasma NT-proBNP, median (range) (pg/ml)	Hazard ratios (95% confidence interval) & p value			
		Unadjusted	Model 1	Model 2	Model 3
NT-proBNP	165.0 (65.7-524.5)	1.0 (1.0-1.0)<br>P = 0.105	1.0 (1.0-1.0)<br>P = 0.838	1.0 (1.0-1.0)<br>P = 0.108	1.0 (1.0-1.0)<br>P = 0.705

Note: Univariable and multivariable cox proportional hazard analysis. Associations between NT-proBNP level and renal outcomes. Model 1 adjusted for baseline age, gender, SBP and the history of CVD. Model 2 adjusted for covariates in model 1 plus e-GFR and proteinuria. Model 3 adjusted for covariates in model 2 plus RPS classfication and IFTA. CI, confidence interval. SBP, systolic blood pressure; CVD, cardiovascular disease; e-GFR, estimated glomerular filtration rate; RPS, renal pathology society glomerular classification; IFTA, interstitial fibrosis tubular atrophy.

Information Classification: General

Supplement table 5. Univariable and multivariable Cox proportional hazard analysis. Associations between baseline renal pathology score and renal outcomes.

	D-score	Hazard ratios (95% confidence interval) & p value		
		Unadjusted	Model 1	Model 2
Q1	≤14	Reference	Reference	Reference
Q2	15-18	3.32 (0.7-15.2)<br>P = 0.122	4.22 (0.9-19.8)<br>P = 0.068	6.16 (0.7-50.1)<br>P = 0.307
Q3	19-21	9.44 (2.1-42.3)<br>P = 0.003	13.2 (2.8-62.6)<br>P = 0.01	12.1 (1.4-106.9)<br>P = 0.025
Q4	22-25	9.49 (1.9-40.1)<br>P = 0.006	12.9 (2.5-66.5)<br>P = 0.002	11.1 (1.1-107.8)<br>P = 0.037

Note: Univariable and multivariable Cox proportional hazard analysis. Associations between NT-proBNP level and renal outcomes. Model 1 adjusted for baseline age and gender. Model 2 adjusted for covariates in model 1 plus e-GFR and proteinuria. CI, confidence interval.

Information Classification: General
